# The impact of COVID-19 on speech–language and hearing professions in low- and middle-income countries: Challenges and opportunities explored

**DOI:** 10.4102/sajcd.v69i2.937

**Published:** 2022-09-09

**Authors:** Katijah Khoza-Shangase, Nomfundo Moroe, Joanne Neille, Anita Edwards

**Affiliations:** 1 Department of Audiology, Faculty of Humanities, University of the Witwatersrand, Johannesburg, South Africa; 2 Department of Speech–Language Pathology, Faculty of Humanities, University of the Witwatersrand, Johannesburg, South Africa; 3 Africa Health Research Institute, Mtubatuba, South Africa

**Keywords:** audiology, COVID-19, teaching and learning, practice, speech–language pathology, clinical research, hearing professions

## Abstract

**Background:**

Since the advent of the coronavirus disease 2019 (COVID-19), the speech–language and hearing (SLH) professions globally have been confronted with novel and unexpected challenges.

**Objective:**

The aim of this article was to explore the impact of COVID-19 on SLH professions in low- and middle-income countries (LMICs) as presented in the Special Issue of the *South African Journal of Communication Disorders* in the year 2022.

**Method:**

Divergent from the standard editorial writing style, this editorial adopted a research approach where a qualitative, descriptive scoping review design was conducted to meet the objectives of the study. Three specific objectives were targeted: (1) exploring the challenges to SLH research, teaching and practice; (2) establishing evidence-based solutions available for these challenges that can be used to improve the professions’ response in the post-pandemic era; and (3) determining the areas that require further investigation, alternative solutions and innovation for improved readiness for future pandemics. A total of 21 manuscripts were reviewed that covered three predetermined themes – research, teaching and practice – that were constructed through a deductive approach as part of the call for papers for the special issue. These manuscripts were from academics, researchers and clinicians from various institutions in LMICs. The review is presented using thematic analysis.

**Results:**

The review raised important challenges, presented under various subthemes, to the three key themes. These challenges reflect on the impact of COVID-19 on the SLH professions in terms of research, teaching, service provision and ethical challenges, as well as its impact on speech language, hearing, swallowing and balance functions. The review also advanced solutions and future directions during and beyond COVID-19.

**Conclusion:**

These findings raise global implications for research, teaching and practice that are not only relevant to the SLH professions.

## Introduction

No country, sector or profession has been spared from the impact of the global health emergency, the coronavirus disease 2019 (COVID-19) pandemic, that has gripped the world since it was first identified and reported in Wuhan, China (World Health Organization [WHO], 2020a, 2020b). Global statistics, as of 27 May 2022, indicate that the total number of confirmed cases in the world has escalated to approximately 528 million, with over 6.28 million deaths. Africa is currently recording 12 122 000 cases, with South Africa registering the highest number of cases at a cumulative total of 3.94 million cases and over 101 000 total official deaths (WHO, 2022). Despite all efforts to curb the spread of the virus and its consequent mortality and morbidity, the reported infection numbers remain high, although the mortality rate is seen to be declining where vaccination roll-out is successful (Cooper, Van Rooyen, & Wiysonge, 2021; WHO, 2022). However, Africa’s vaccination drive has confronted numerous challenges, including resource constraints as well as vaccine access and hesitancy, with South Africa at the end of the fifth wave of the pandemic in this second quarter of the year. This reality, therefore, has necessitated continued use of nonpharmaceutical prevention and control of the COVID-19 infection measures, such as wearing of face coverings and physical distancing, in an effort to save lives and livelihoods, while preserving the health and safety of health care practitioners and their patients and students (Beach et al., 2020; WHO, 2022; Wieten, Burgart, & Cho, 2020), with these regulations very recently (June 2022) having been completely scrapped by the South African National Department of Health. The implementation of these measures has presented challenges to the provision of services within the scope of practice of the speech–language and hearing (SLH) professions, including clinical practice, teaching and training, as well as research (Khoza-Shangase et al. 2021).

A considerable aspect of the measures aimed at preventing the spread of COVID-19 concentrated on the application of different public health measures for reducing transmission (Kamerlin & Kasson, 2020). These prevention measures have been classified as ‘*suppressive* approaches, which aim to arrest transmission, and *mitigation* approaches, which aim to slow spread and shield vulnerable populations without truncating transmission’ (Kamerlin & Kasson, 2020, p. 1). Strategies such as ventilation, wearing of face coverings and physical distancing are aimed at curbing the spread of the virus, because close contact has been identified as a mode of virus transmission via droplet and airborne routes through respiratory activities (Sun & Zhai, 2020). Such strategies of reducing transmission, especially within the SLH professions where the patient population falls under the vulnerable groups (Kuy et al., 2020), highlighted noteworthy challenges while raising the need for innovative solutions in clinical practice, training and research in these fields. Thus, there is the need for the current Special Issue of *South African Journal of Communication Disorders* (SAJCD) in 2022 on the impact of COVID-19 in SLH professions in low- and middle-income countries (LMICs).

Besides the obvious impact on the ability to provide clinical care for persons with communication impairments who rely on face-to-face communication (Tohidast, Mansuri, Bagheri, & Azimi, 2020), as well as the potential cognitive-communicative, hearing and balance impairments caused by COVID-19 (Fancello et al., 2021; Jacob, Flannery, & Mostert, 2020; Ramage, 2020), evidence also suggests that the use of masks majorly impacts communication in at least two ways. The wearing of masks and adherence to physical distancing is believed to affect the scope of SLH practice by (1) reducing important features such as visual cues (Chodosh, Weinstein, & Blustein, 2020; Homans & Vroegop, 2021; Kataoka, Maeda, Sugaya, Omichi, & Kariya, 2021) and (2) lowering speech sound intensity and clarity, which affects individuals who depend on this compensatory strategy, such as the hearing impaired (Saunders, Jackson, & Visram, 2021; Ten Hulzen & Fabry, 2020). Research indicates that masks cause a 3 dB – 4 dB (medical masks) reduction to approximately 12 dB (N95 mask) reduction in sound intensity and a low-pass filter effect attenuating 2000 Hz – 7000 Hz of the speaker’s voice (Goldin, Weinstein, & Shiman, 2020).

Exploration of the impact of COVID-19 in LMICs has substantial implications for SLH professionals. It archives challenges encountered within these resource-constrained contexts. Furthermore, it documents innovative solutions that were applied to overcome these challenges. This will allow for careful future planning, not only for future public health emergencies, but also for re-imagined service provision in these contexts. This exploration further highlights challenges of capacity versus demand as far as SLH human resources are concerned, in contexts where this is an additional pressure for speech, language, swallowing, hearing and balance care (Khoza-Shangase, 2021; Pillay, Tiwari, Kathard, & Chikte, 2020). Within the South African context, such careful future planning, as well as re-imagined SLH research, teaching and practice, requires an evidence base that can help facilitate service delivery models that are culturally, linguistically and contextually compatible with the population and context (Khoza-Shangase, 2020). It is against this backdrop that the authors, Professors Katijah Khoza-Shangase, Nomfundo Moroe and Joanne Neille, all of whom are academics and researchers in the Departments of Audiology and Speech–language Pathology at the University of the Witwatersrand, initiated the concept of, and acted as guest editors, on this Special Issue of the SAJCD, entitled ‘The impact of COVID-19 in SLH professions in low and middle-income countries: Challenges and opportunities explored’, by inviting research papers from academics, clinicians and researchers.

The current article therefore serves as both the editorial for the Special Issue and original research in its own right.

## Method

### Aim

Instead of the standard editorial that provides an overview of papers included in the Special Issue of SAJCD in 2022, the editorial team adopted a research approach that aimed at exploring the collated views and findings on the impact of COVID-19 on SLH professions in LMICs, as reported in papers accepted for publication in the current Special Issue of the SAJCD, with very specific objectives.

### Objectives

This study had the following objectives:

1. to explore the challenges to SLH research, teaching and practice during the COVID-19 pandemic
2. to describe evidence-based solutions for SLH research, teaching and practice in the post-pandemic era
3. to identify the issues that require further investigation for improved readiness for future pandemics.

### Research questions

The specific research questions asked by the authors were as follows:

1. What were the challenges to SLH research, teaching and practice during a pandemic like COVID-19 reported by authors of the articles in the SAJCD Special Issue (2022)?
2. What evidence-based solutions were recommended for SLH research, teaching and practice that can be used to improve the professions’ response in the post-pandemic era?
3. What areas of research require further investigation and alternative solutions and innovation for improved readiness for future pandemics?

### Study design

Because of the fact that a limited comprehensive body of literature existed on COVID-19 and its potential impact on SLH in LMICs at the time of the Special Issue, as well as the fact that the paper was exploring evidence specific to time (during COVID-19), location (LMICs), source (peer-reviewed articles included in the Special Issue) and origin (academics, researchers and clinicians), a qualitative descriptive scoping review design was deemed an appropriate approach to adopt (Peters et al., 2022). This design allowed for an overview of the complex impact that the COVID-19 public health emergency had on the SLH professions. The research design was chosen to facilitate recommendations for improved health care decision-making processes and quality of health care services in the SLH professions (Bae, 2014; Butina, 2015; Green, Johnson, & Adams, 2006).

### Sources of data

Sources of data for the current study included all manuscripts accepted for publication in the Special Issue of the SAJCD in 2022 after a blinded peer review process. A public call for submissions to the Special Issue of SAJCD in 2022 was made and the guest editors encouraged those in the SLH professions in LMICs known to be involved in aspects related to the topic from among academics, researchers and clinicians to submit manuscripts. The Special Issue aimed to collate current contextually relevant and responsive evidence that fills the gap in innovative practices and re-imagines future practice in SLH professions, as well as different models of service delivery. The goal of the Special Issue was to reflect on the impact of COVID-19 on the SLH professions in terms of research, teaching, service provision and ethical challenges as well as its impact on speech–language, hearing, swallowing and balance functions.

### Selection criteria

All manuscripts accepted for publication in the Special Issue of SAJCD in 2022 were included in this qualitative narrative review. For inclusion in the Special Issue, manuscripts had to meet the following criteria:

1. must be written in English
2. consider the impact of COVID-19 on one or more of the following areas: impact on training of SLH professions; impact on research on SLH professions; impact on speech–language, hearing, swallowing and balance functions; contextual factors influencing SLH professions’ clinical response to COVID-19; policy and legislation in SLH professions’ response to COVID-19; and impact on service delivery by SLH professionals.

### Data analysis

A narrative thematic data analysis process was used, consisting of the following stages (Creswell & Creswell, 2017):

1. arrangement and organisation of the data
2. gaining an overall appreciation of the information
3. identifying themes aligned to the research questions
4. interpretation of the data.

The analysis of data also included the qualitative narrative review approach of reflection on relevant evidence of the authors’ findings in the 21 articles included in the Special Issue (Butina, 2015). Analysis was further enhanced by using the framework of ‘ask – acquire – appraise – apply – assess’ or the 5A process, proposed by Green et al. (2006) to facilitate critique and to summarise the contents of the findings of the articles.

Succinctly summarised data relevant to the specific objectives of the study were extracted and are presented in Table 1 and Table 2. Readers are referred to the individual specific articles in the Special Issue of SAJCD in 2022 for more specific details on the findings, as these could not be detailed in this article. The tables reflect the following information extracted from the reviewed manuscripts:

- author(s) and the year of publication
- title of the study
- challenges reported in terms of research, teaching and practice (Table 1)
- solutions and future research directions recommended in terms of research, teaching and practice (Table 2).

**TABLE 1:** Summary of studies included in the scoping review documenting challenges presented by the COVID-19 pandemic to practice, teaching and research.

Authors	Title	Challenges
1. Khoza-Shangase, Moroe and Sebothoma (2022)	Conducting clinical research in the era of the COVID-19 pandemic: Challenges and lessons for speech–language pathology and audiology research	**Practice:** Not applicable
		**Teaching:** Not applicable
		**Research:** Lack of processes to balance priority, speed and quality of research; need for flexibility in research protocols and designs; participant recruitment and participation; challenges of obtaining informed consent remotely; information and communications technology challenges; challenges with interventions; challenges with data capturing; analysis and storage; challenges with sharing and publishing findings.
2. Khoza-Shangase (2022a)	Cochleovestibular findings linked to COVID-19: A scoping review for clinical care planning in South Africa	**Practice**: Difficulty determining whether COVID-19 causes cochleovestibular disorders; difficulty determining whether masks mask cochleovestibular disorders; uncertainty and inconsistency regarding the underlying pathophysiology of the cochleovestibular symptoms in COVID-19-positive patients; epidemiological correlations and bio-pathological mechanisms involved in cochleovestibular presentation of COVID-19 require further investigations; practitioners were cautioned regarding the assessment and management of patients presenting with unexplained cochleovestibular symptoms during the pandemic.
		**Teaching:** Not applicable
		**Research:** Poor quality of studies documenting vestibulocochlear disorders during this time, especially in terms of research design.
3. Khoza-Shangase (2022b)	In pursuit of increasing the application of tele-audiology in South Africa: COVID-19 puts on the alert for patient site facilitator (PSF) training	**Practice:** Lack of sufficient knowledge and skills of telepractice by audiologists; lack of access to the required infrastructure and technology by both patients and clinicians; lack of good Internet connectivity, reimbursement and licensure barriers; lack of reliable electricity supply; diversity in the range of PSFs, which has implications for the nature and the type of training.
		**Teaching:** Lack of regularisation, standardisation and structure in current PSF training programmes.
		**Research:** Not applicable
4. Nagdee, Sebothoma, Madahana, Khoza-Shangase and Moroe (2022)	Simulations as a mode of clinical training in healthcare professions: A scoping review to guide planning in speech–language pathology and audiology during the COVID-19 pandemic and beyond	**Teaching:** Challenges with accommodating growing student numbers in clinical platforms; skill retention in simulated learning is limited, implementation of simulations as a mode of clinical training requires regulatory approval. Teaching: Costs associated with providing clinical training. Access to in-person training is required in addition to simulation training – simulation cannot be done in isolation.
5. Sebothoma and Khoza-Shangase (2022)	Middle ear status – Structure, function, and pathology: A scoping review on middle ear status of COVID-19-positive patients	**Practice:** There is limited insight into how middle ear function can be measured using immittance measures; difficult to make a comparison of the proportion of conductive hearing loss with studies from the general population and/or those with participants infected with other viruses.
		**Teaching:** Not applicable
		**Research:** Poor quality of studies.
6. Madahana, Khoza-Shangase, Moroe, Nyandoro and Ekoru (2022a)	Application of machine learning approaches to analyse student success for contact learning and emergency remote teaching during the COVID-19 era in speech–language pathology and audiology	**Practice:** Not applicable
		**Research:** Not applicable
		**Teaching:** Challenges moving to online platforms; power interruptions, stable Internet connections, lack of peer interactions, lack of conducive learning environments, lack of e-tech facilities and lack of technical skills; budget costs; student funding.
7. Madahana, Khoza-Shangase, Moroe, Mayombo, Nyandoro and Ekoru (2022b)	A proposed artificial intelligence-based real-time speech-to-text-to-sign language for South African official languages translator for the COVID-19 era and beyond: In pursuit of solutions for the hearing impaired	**Practice:** Lack of utilisation of artificial intelligence for clinical application; real-time captioning currently only available in English; depending on one’s accent, the translation of English speech-to-text may also not be accurate.
		**Teaching**: Not applicable Research: Lack of research into artificial intelligence as a machine learning option, especially for individuals with hearing impairment; lack of urgency for developing artificial intelligence to assist individuals with hearing impairment; lack of research in the African continent on artificial intelligence and machine learning in SLH.
8. Naidoo et al. (2022)	Speech–language therapy educator reflections on the planning and implementation of education and training during the COVID-19 pandemic	**Practice:** Not applicable
		**Teaching:** Emergency remote teaching and learning (ERTL) had to be introduced. This was daunting for educators, however, because of limited knowledge and skills of ERTL, which led to uncertainty among educator participants in the study. Although the training provided was useful, it lacked theoretical coherence and focused more on knowledge than on skills. Although similar challenges were experienced by all students, first-year students especially experienced more challenges because of lack of timely institutional support. Educators struggled to cope with the additional demands that came with the adoption of a student-centred approach – increased workload.
		**Research:** Not applicable
9. Balton, Vallabhjee and Pillay (2022)	When uncertainty becomes the norm: The Chris Hani Baragwanath Academic Hospital’s Speech Therapy and Audiology Department’s response to the COVID-19 pandemic	**Practice:** The response of the South African health care system showed that the needs of vulnerable populations were not accounted for when developing the public health response to a pandemic (policies and regulations).
		**Teaching:** Not applicable
		**Research:** Not applicable
10. McAllister et al. (2022)	Telesupervision and online case-based learning can support successful learning outcomes for speech and language therapy students in Vietnam	**Practice:** Cessation of face-to face-teaching because of COVID-19. Online learning and simulation were rarely used in university programmes prior to the COVID-19 pandemic. Students had smart phones but not necessarily laptops, and Internet with good bandwidth was often limited in their homes.
		**Teaching:** Not applicable
		**Research:** Not applicable
11. Tar-Mahomed and Kater (2022)	The perspectives of speech–language pathologists: Providing teletherapy to patients with speech, language and swallowing difficulties during a COVID context	**Practice:** There are various barriers to the implementation of telemedicine solutions in the public sector and telemedicine in general in South Africa, for example, challenges with technology and connectivity. Speech-language pathologists (SLPs) experience a variety of barriers and disadvantages while using teletherapy. SLPs felt like therapy was less personal and that there was a lack of rapport building (clinician–client relationships). Issues of access differ across the private and public sector SLPs for both the clients and the SLPs. Load-shedding (national electricity supply interruptions), data costs, access and connection difficulties, as well as limited access to devices and limited technological abilities. Experiences differ in terms of patient participation, outcomes and the multidisciplinary team, as well as about the different disorders seen.
		**Teaching:** Not applicable
		**Research**: Lack of research on the use of teletherapy for patients with speech, language and swallowing difficulties after a stroke from an SLP’s point of view.
12. Khatib and Hlayisi (2022)	Rehabilitation in the time of COVID-19: Is a hybrid telehealth approach the new norm?	**Practice**: Need for rapid uptake of tele-audiology because of COVID-19 to adhere to mandatory health and safety protocols. Cost and workload challenges of a hybrid model. Challenges including technology limitations (e.g. Internet-capable device access and technological compatibility of older hearing aids), patient readiness and reliability of some online assessments.
		**Teaching:** Not applicable
		**Research:** Not applicable
13. Hlayisi (2022)	Increasing unemployment rate among health professionals: Will there be jobs for newly graduated South African audiologists post-COVID-19?	**Practice:** Challenges with provision of hearing care services because of increased unemployment rate – worsened by COVID-19 and affecting health care professionals. For the employed audiologists, the most common workplace challenges in descending order were remuneration, lack of resources, workload, work environment, working hours and interprofessional relationships.
		**Teaching:** Not applicable
		**Research:** Not applicable
14. Achmat and Gerber (2022)	Challenges to infection control in early communication intervention: A scoping review	**Practice:** Provision of early communication intervention in the context of preventing the spread of COVID-19 – using personal protective equipment (PPE) and infection prevention and control (IPC) measures. *Challenges to establish IPC measures within early intervention*: the nature of care and behaviour of young children; infrastructure and system challenges; a lack of standard IPC protocols (e.g. differences in IPC measures between medical and nonmedical settings); poor IPC compliance among health care workers (HCWs), patients and caregivers; and a lack of IPC training for staff and caregivers. *Challenges to conduct early communication intervention (ECI) when utilising IPC measures:* difficulty targeting prelinguistic skills; difficulty providing facial cues; PPE is impersonal; over-compliant caregivers; special needs populations are not considered.
		**Teaching:** Not applicable
		**Research:** Not applicable
15. Barber and Sher (2022)	Exploring the online learning experience of first-year speech–language pathology students in a Johannesburg-based university	**Practice:** Not applicable
		**Teaching:** COVID-19 pandemic as a disruptor of traditional education. South Africa’s history of marginalised populations who have not been provided with equitable educational services. Varied demographics of students requiring different types of support to succeed.
		**Research:** Not applicable
16. Karrim, Flack, Naidoo, Beagle and Pontin (2022)	The experiences of speech–language therapists providing telerehabilitation services to children with autism spectrum disorder	**Practice:** Challenges with criteria that speech–language therapists (SLTs) use when recommending telerehabilitation for children with autism spectrum disorder, the technical skills required, strategies used by SLTs, the restrictions encountered when conducting telerehabilitation. Challenges with restrictions in policies regarding the use of telerehabilitation for assessment and treatment, prior knowledge, load-shedding, behavioural management and conducting assessments.
		**Teaching:** Not applicable
		**Research:** Not applicable
17. Masuku and Mupawose (2022)	Students’ experiences of using a writing intense programme to facilitate critical thinking skills on an online clinical training platform: A pilot study	**Practice:** Not applicable
		**Teaching**: Challenges with in-person addressing of knowledge gaps, clinical reasoning skills; clinical competencies and academic writing skills as part of clinical training because of COVID-19 restrictions.
		**Research:** Not applicable
18. Masuku, Khumalo and Shabangu (2022)	The effects of COVID-19 on the rehabilitation of persons with aphasia: A scoping review	**Practice:** Five themes: (1) negative impact on rehabilitative care, (2) telehealth and its limitations, (3) impact on social participation, (4) compromised caregiver involvement and (5) mental health challenges.
		**Teaching:** Not applicable
		**Research**: There is documented evidence of the rippling effects of COVID-19 on persons with disabilities. However, not much is known about the impact of COVID-19 on persons with aphasia, specifically rehabilitation.
19. Coutts, Neille and Louw (2022)	Feeding practices in public hospitals’ neonatal intensive care units: An exploration into the ways in which COVID-19 affected best practice in Gauteng	**Practice:** The South African health care system has a multitude of pre-existing challenges prior to the onset of the COVID-19 pandemic, ranging from reduced number of staff, lack of resources and intensive care units being at over-capacity both in the adult and paediatric populations. Physical distancing was a significant challenge, resulting in mothers and other HCWs being given restricted access to the neonatal intensive care unit (NICU), thus negatively affecting the ability to provide adequate feeding practices – leading to increased anxiety for the mothers and mental health challenges for the HCWs when feeding these at-risk infants.
		**Teaching:** Not applicable
		**Research:** Not applicable
20. De Andrade and Landman (2022)	*‘*All of a sudden, you know, you can’t go to these services, because of the risk of infection’: Audiological service considerations at residential care homes for older persons in Johannesburg during COVID-19 restrictions	**Practice:** Seven themes: (1) audiological services were interrupted because of COVID-19; (2) other health services were prioritised over audiological services because of COVID-19; (3) COVID-19 mask-wearing (and physical distancing) adversely impacted communication with older persons in residential care homes; (4) communication had to be adjusted for residents who have hearing loss during COVID-19 restrictions; (5) COVID-19 increased the sense of isolation for residents; (6) financial constraints limited the provision of audiological services; and (7) COVID-19 impacted the confidence in hospital attendance for audiological service provision.
		**Teaching:** Not applicable
		**Research:** Not applicable
21. Khan, Mthembu, Narothan, Sibisi and Vilane (2022)	Health sciences student’s perception of the communicative impacts of face coverings during the COVID-19 pandemic at a South African university	**Practice:** Challenges envisaged in practice included frequent communication breakdowns, inability to connect and build trust between patient and practitioner and communicating in noisy environments.
		**Teaching:** Not applicable
		**Research:** Masks and shields can impair communication, facial expressions, affect sound transmission and remove visual cues, making hearing speech challenging. Mask-wearing requires more listening effort and raises feelings of anxiety when communicating. Challenging to read emotions, such as sadness or unhappiness, when someone wears a mask. Challenges worse when listening to an unfamiliar person.

COVID-19, coronavirus disease 2019; ERTL, emergency remote teaching and learning; HCWs, health care workers; SLH, speech–language and hearing; SLT, speech–language therapist.

**TABLE 2:** Summary of studies included in the scoping review documenting solutions and future research directions for COVID-19.

Authors	Title	Solutions and future research directions
1. Khoza-Shangase et al. (2022)	Conducting clinical research in the era of the COVID-19 pandemic: Challenges and lessons for speech–language pathology and audiology research	**Practice:** Not applicable
		**Teaching:** Innovative research methods should be included in the curriculum.
		**Research:** New approaches to research, development of research infrastructure and work force, opportunities for remote working practices; improved use of ICT for research processes.
2. Khoza-Shangase (2022a)	Cochleovestibular findings linked to COVID-19: A scoping review for clinical care planning in South Africa	**Practice**: Need for careful audiological assessment, as well as polymerase chain reaction testing for COVID-19 diagnosis in patients presenting with sudden unexplained cochleovestibular symptoms during COVID-19.
		**Teaching:** Not applicable
		**Research:** Need for more rigorous research designs, including longitudinal studies to establish the effect of COVID-19 on cochleovestibular functioning
3. Khoza-Shangase (2022b)	In pursuit of increasing the application of tele-audiology in South Africa: COVID-19 puts on the alert for patient site facilitator training	**Practice:** Potential role of PSFs in tele-audiology or telepractice with the potential of enhancing contextual relevance; creation of job opportunities where paraprofessionals are utilised in task-shifting roles where they serve as PSFs; considerations for asynchronous teletherapy; need for quality standards and best-practice models to be maintained.
		**Teaching:** Teaching requires careful interrogation of this core aspect of tele-audiology. Need for intensive training of PSFs, especially about asynchronous service delivery; option for ongoing training of PSFs during synchronous tele-audiology; considerations of content and practical training of PSFs.
		**Research:** Opportunities for research to be more contextually relevant and responsive; linguistic and cultural diversity considerations can be improved.
4. Nagdee et al. (2022)	Simulations as a mode of clinical training in healthcare professions: A scoping review to guide planning in speech–language pathology and audiology during the COVID-19 pandemic and beyond	**Practice:** Not applicable
		**Teaching:** Simulations mitigate risk of compromising patient safety; support the shift towards competency-based education; facilitate basic knowledge acquisition; improve clinical skills and enhance clinical decision-making. Simulations allow for core competencies to be achieved and enhance knowledge.
		**Research:** Research into the use of simulations in SLH – including its effectiveness. This must be from both students’ and educators’ perspectives.
5. Sebothoma and Khoza-Shangase (2022)	Middle ear status – Structure, function, and pathology: A scoping review on middle ear status of COVID-19-positive patients	**Practice:** Need to determine whether COVID-19 causes structural damage to the middle ear.
		**Teaching:** Not applicable
		**Research:** Additional research is required into whether COVID-19 affects middle ear function.
6. Madahana et al. (2022a)	Application of machine learning approaches to analyse student success for contact learning and emergency remote teaching during the COVID-19 era in speech–language pathology and audiology	**Practice:** The results of the study indicated: a need for robust technical infrastructure to support online learning a need for a paradigm shift to inclusion of artificial intelligence (AI) and machine learning (ML) as the country transitions towards the fourth industrial revolution.
		**Teaching:** Flexibility in terms of access to academic material; need for robust technical infrastructure.
		**Research:** Research into AI and ML in SLH.
7. Madahana et al. (2022b)	A proposed artificial intelligence-based real-time speech-to-text-to-sign language for South African official languages translator for the COVID-19 era and beyond: In pursuit of solutions for the hearing impaired	**Practice:** Current technologies being developed in South Africa should include AI and ML concepts in their design of solutions that are targeted towards bridging communication between the normal hearing individuals and those with hearing loss.
		**Teaching:** AI and ML should be included in the training of students – how? What should specifically be included in student training? Research: Lack of research into AI as an ML option, especially for individuals with hearing impairment; lack of urgency for developing AI to assist individuals with hearing impairment; lack of research in the African continent on AI and ML in SLH.
8. Naidoo et al. (2022)	Speech–language therapy educator reflections on the planning and implementation of education and training during the COVID-19 pandemic	**Practice:** Opportunity for staff to enhance their digital literacy means improved confidence and competence, thus implications for continued use of online teaching, learning and assessment to some degree, regardless of return to face-to-face teaching. The use of combined technology supported digital learning with face-to-face contact within a hybrid model.
		**Teaching:** Online platforms have also facilitated more collaboration between students, between students and educators and between educators within the department and internationally. Opportunities to support students through social media platforms could result in students receiving more immediate and individual support.
		**Research:** Not applicable
9. Balton et al. (2022)	When uncertainty becomes the norm: The Chris Hani Baragwanath Academic Hospital’s Speech Therapy and Audiology Department’s response to the COVID-19 pandemic	**Practice:** Importance of adaptability, becoming comfortable with uncertainty and maintaining open and transparent communication. Consultation and collaboration within various levels of the health care system is critical in responding to the needs of patients. Commitment to compassionate leadership and staff well-being is crucial.
		**Teaching:** Not applicable
		**Research:** Not applicable
10. McAllister et al. (2022)	Telesupervision and online case-based learning can support successful learning outcomes for speech and language therapy students in Vietnam	**Practice:** Not applicable
		**Teaching:** Telesupervision where international therapists, working remotely and in partnership with local therapists, supervise on-site placements for students. Online case-based discussions as part of clinical training. Online student group discussions, using case simulations with videos or avatars. All the above points do not exclude face-to-face placements (direct experiences with patients).
		**Research:** Not applicable
11. Tar-Mahomed and Kater (2022)	The perspectives of speech–language pathologists: Providing teletherapy to patients with speech, language and swallowing difficulties during a COVID context.	**Practice:** Paying attention to the following factors is important: socio-economic status, culture, education, income, as well as access to information and communication technologies, their associated infrastructure and a stable electricity supply.
		**Teaching:** Not applicable
		**Research:** Not applicable
12. Khatib and Hlayisi (2022)	Rehabilitation in the time of COVID-19: Is a hybrid telehealth approach the new norm?	**Practice:** Use of a hybrid telerehabilitation approach that can yield (1) high compliance, (2) good clinical benefit, (3) positive participant experience and (4) low costs (cost-effective).
		**Teaching:** Not applicable
		**Research:** Large-scale studies required on this in LMICs for evidence-based implementation.
13. Hlayisi (2022)	Increasing unemployment rate among health professionals: Will there be jobs for newly graduated South African audiologists post-COVID-19?	**Practice:** Not applicable
		**Teaching:** Not applicable
		**Research:** Research into hearing health care human resource policies and planning, hearing health care labour market needs and capacity as well as hearing health care service delivery and potential for growth in the South African context after COVID-19.
14. Achmat and Gerber (2022)	Challenges to infection control in early communication intervention: A scoping review	**Practice:** Not applicable
		**Teaching:** Not applicable
		**Research:** Further research is needed to determine whether IPC measures and/or ECI strategies can be modified to collectively maintain their effective implementation. For example, investigations on the effectiveness of different types of PPE which presents the least interference to intervention, such as wearing a face shield with a transparent cover instead of a face mask that obstructs visual cues.
15. Barber and Sher (2022)	Exploring the online learning experience of first-year speech–language pathology students in a Johannesburg-based university.	**Practice:** Not applicable
		**Teaching:** Hybrid models of teaching yield positive outcomes (e.g. prerecorded lectures, weekly engagement tasks, live lectures and Microsoft Teams breakout rooms), with prerecorded lectures yielding best and live lectures yielding least positive responses. A period of adjustment and supportive strategies required. ‘Contextual influences’ and ‘coping mechanisms and strategies’ must be taken cognisance of. ‘Personal challenges’ and ‘building resilience’ must be attended to.
		**Research:** Not applicable
16. Karrim et al. (2022)	The experiences of speech–language therapists providing telerehabilitation services to children with autism spectrum disorder	**Practice:** Telerehabilitation used to provide assessment and therapy during the COVID-19 pandemic lockdowns as an alternative method of service delivery. Assessment and treatment strategies include synchronous and asynchronous methods, family collaboration, social stories, frequent breaks and interactive sessions. Benefits far outweigh the challenges encountered.
		**Teaching:** Not applicable
		**Research:** Not applicable
17. Masuku and Mupawose (2022)	Students’ experiences of using a writing intense programme to facilitate critical thinking skills on an online clinical training platform: A pilot study	**Practice:** Not applicable
		**Teaching**: Synchronous and asynchronous writing-intensive clinical programme to address knowledge gaps, clinical competencies and academic writing skills to mitigate the challenges presented by COVID-19 on clinical training. Use of simulation approach that incorporates case studies and writing intensives to develop clinical reasoning skills of second-year students (new to clinical practice). Enhanced clinical report-writing skills and integration of theory with practice.
		**Research:** Not applicable
18. Masuku et al. (2022)	The effects of COVID-19 on the rehabilitation of persons with aphasia: A scoping review	**Practice:** Prioritisation of mental health services for persons with aphasia and their caregivers during a pandemic.
		**Teaching:** Not applicable
		**Research**: Investigate innovative ways in which aphasia rehabilitation and conversational support programmes can still be made accessible to persons with aphasia despite the limitations brought about by COVID-19.
19. Coutts et al. (2022)	Feeding practices in public hospitals’ neonatal intensive care units: An exploration into the ways in which COVID-19 affected best practice in Gauteng.	**Practice:** Neonatal intensive care units (NICUs) need an increase in HCWs to provide the services needed by the number of infants in this setting when it comes to adequate feeding practices of these at-risk infants. Implementation of a more transdisciplinary approach to the management of feeding within the NICUs during these circumstances and how to better utilise mothers within the NICU environment to assist with feeding.
		**Teaching** Not applicable
		**Research:** Investigations on the longer-term effects that COVID-19 has had on this setting, the HCWs, infants and families.
20. De Andrade and Landman (2022)	*‘*All of a sudden, you know, you can’t go to these services, because of the risk of infection’: Audiological service considerations at residential care homes for older persons in Johannesburg during COVID-19 restrictions	**Practice:** Need to balance audiological needs with other health needs. Adapted strategies need to be considered to support communication considering COVID-19 precautions so that communication difficulties do not exacerbate lockdown isolation (e.g. use of transparent masks, reprogramming of hearing aids to account for sound attenuation because of masks, etc.).
		**Teaching:** Not applicable
		**Research:** Not applicable
21. Khan et al. (2022)	Health sciences students’ perception of the communicative impacts of face coverings during the COVID-19 pandemic at a South African university	**Practice:** Not applicable
		**Teaching:** More education and training of students in health science disciplines and possibly the wider university community on the use of face coverings and shields and communication impact. Education and information on the effects that different face masks and shields have on hearing and communication and the impact on interpersonal communication, ways in which communication breakdowns can occur with face masks and shields and how to mitigate these.
		**Research:** Investigations into how patients with hearing loss receive appropriate health care information and how they adhere to instructions given by the audiologist when both are wearing masks and face coverings. Research the interaction of cultural, language and dialects diversity and mask-wearing. Impact of mask-wearing on teaching and learning.

SLH, Speech–language and hearing; LMIC, low- and middle-income countries; ICT, information and communications technology; PSF, patient site facilitator; IPC, infection prevention and control; PPE, personal protective equipment; ECI, early communication intervention; NICU, neonatal intensive care unit; HCW, health care worker.

The analysis was conducted by three of the authors, who paid particular attention to the information that offered insight into the stated research questions (Butina, 2015). Manuscript (member)-checking (the SAJCD’s independent review process), confirmability between all researchers and transferability and dependability were all carried out to promote credibility.

#### Data management

The guest editors and the SAJCD editor-in-chief, as well as the administrative staff working for the publishing company, had access to the submitted manuscripts and the blinded peer review process used by the publishing company. An electronic audit trail was ensured by saving all the manuscripts reviewed and notes for data collection and analysis in password-protected information and communications technology (ICT) systems (Butina, 2015; Creswell & Creswell, 2017).

### Ethical considerations

The nature of the current study based on secondary data (articles in the Special Issue of SAJCD in 2022) precluded the need for ethical clearance. However, this scoping review followed all ethical standards for a study that does not involve direct contact with human or animal subjects. This included reflexivity and informed subjectivity, audience-appropriate transparency and purposefully informed selective inclusivity (Suri, 2020).

## Results and discussion

This Special Issue contains 21 papers that accurately responded to the specified call and thus met the eligibility criteria. These papers are depicted in Table 1 and Table 2. These papers, although unequally, addressed the three key themes that formed part of the open call for papers for the SAJCD Special Issue (2022): (1) research, (2) teaching and (3) practice in SLH in LMICs. These three themes were established through a deductive approach. Nuanced analysis of the focus on the evidence provided by the papers revealed a clear preponderance of papers focusing on practice challenges, possible solutions and future directions for clinical practice in SLH (11 of the 21 papers). Six papers specifically focused on teaching and learning challenges as well as solutions and future directions.

The papers, which are all published in the SAJCD Special Issue (2022), were all listed by the authors as reviews or original research articles. The papers are from LMICs, as per the open call. South Africa is the most represented country, possibly because the journal is owned and supported by the South African Speech Language and Hearing Association and is therefore most known to South African professionals and researchers. Published articles are from more than half (four of seven) of the South African universities where SLH training occurs. The reader is referred to the Special Issue of SAJCD in 2022 for details from individual articles.

Inductive thematic analysis findings are presented and discussed under the specific study objectives, covering all three predetermined major themes, under specifically labelled subtitles.

### Challenges to speech-language and hearing research, teaching and practice during the COVID-19 pandemic

#### Research

The inductive thematic analysis of the extracted data, as presented in Table 1 and Table 2, found that the challenges encountered by SLH researchers during the COVID-19 pandemic could be categorised into three subthemes: (1) paucity in research, (2) a lack of rigorous scientific standards as reflected in the research design and (3) adherence to cumbersome, inflexible and bureaucratic processes for research from research conceptualisation to publishing.

The included papers highlighted a general lack of research during COVID-19 as well as limited research on COVID-19 and its impact on SLH professions. Furthermore, solutions towards provision of services during this public health emergency were also identified as lacking (Barber & Sher, 2022; Khoza-Shangase, 2022a; Khoza-Shangase et al., 2022; Madahana et al., 2022b; Tar-Mahomed & Kater, 2022;). For example, Madahana et al. (2022b) lamented on the paucity of evidence as well as focused research into artificial intelligence (AI) as a machine learning (ML) option, particularly for the hearing-impaired population. This gap is especially pronounced in the African continent. Such a lacuna became intensely obvious during the COVID-19 pandemic, where the use of masks negatively impacted communication. Consequently, a need for AI for clinical application as well as real-time speech captioning in African languages to assist hearing-impaired individuals was highlighted.

Additionally, Tar-Mahomed and Kater (2022) raised another challenge regarding the lack of research on the utilisation of telepractice with SLH patients. Their research focused on evidence for the use of this modality in the provision of therapy to patients with speech, language and swallowing difficulties after a stroke. For this Special Issue of SAJCD in 2022, no submissions of manuscripts on research into any other areas of communication disorders, many of which must have become relevant during the pandemic, were received.

Moreover, Barber and Sher (2022) identified a challenge with limited research evidence into understanding how to facilitate throughput of SLH students during the pandemic. In their review, Khoza-Shangase et al. (2022) performed a scoping review specifically on challenges encountered by health care researchers in conducting research during the COVID-19 pandemic, while highlighting lessons learnt for SLH research. Their review, which echoes challenges also reflected in the other articles included in the SAJCD Special Issue (2022), found the following: (1) an absence of processes that can be used to balance quality, priority and speed of research conducted during this time; (2) an overall need for research protocols and designs to be flexible during the pandemic, as well as the importance of this flexibility in participant recruitment and participation; (3) difficulties with securing informed consent from research participants remotely – where remote working had become the standard practice; (4) ICT challenges where telefunctioning had to be adopted because of a need to adhere to COVID-19 regulations; (5) significant difficulties with provision of research interventions during the research; (6) challenges with data capturing, analysis and storage; and (7) challenges with sharing and publishing research findings. These challenges seem to have an influence on the amount, type and quality of research conducted, produced and shared (Khoza-Shangase, 2022a; Khoza-Shangase et al., 2022). Khoza-Shangase (2022a), for example, specifically highlighted the lack of rigorous scientific standards as reflected in the research designs in studies on cochleovestibular features of COVID-19. The above-mentioned challenges are important to archive to allow for anticipatory strategies to be put in place for future planning.

#### Teaching and learning

As far as teaching and learning challenges during the COVID-19 pandemic were concerned, over and above the impact that masks and shields had on communication (Khan et al., 2022), four themes emerged from the papers reviewed. Khan et al. (2022) highlight the impact of masks and shields as obscuring facial expressions, affecting sound transmission, creating a barrier to accessing visual cues, making hearing speech challenging and listening more effortful and thus increasing feelings of anxiety when communicating during teaching and learning activities. These challenges would obviously not be experienced when teaching occurs online, as shields and masks are not utilised in that modality. Therefore, the four themes that emerged were (1) a general lack of support for students, (2) heightened gaps in skills and knowledge, (3) limited knowledge and skills of educators for emergency remote teaching and learning (ERTL) and (4) exceptionally increased workload for educators. Coronavirus disease 2019 presented unprecedented challenges with traditional methods of teaching and learning, and these required innovative approaches and unconventional solutions. However, institutions of higher learning faced challenges with providing timely support for students to be able to navigate and cope with these institutional changes. Naidoo et al. (2022), in their speech–language therapy educator reflections on the planning and implementation of clinical education during the COVID-19 pandemic, reported that teaching and learning challenges were common across all students but were, however, more pronounced with first-year students. Barber and Sher (2022) observed that the challenges to teaching and learning indicated the need for varied types of support for students from different demographics to succeed. This timely institutional support that students require to be able to traverse the complex institutional changes was deemed crucial during the pandemic. This was particularly because challenges with in-person targeting of gaps in knowledge, clinical reasoning skills and competencies, as well as academic writing skills as part of clinical training, were impacted by COVID-19 restrictions (Masuku & Mupawose, 2022).

Moreover, as far as teaching and learning challenges were concerned, COVID-19-induced ERTL did not only create challenges for students, but for educators as well. Key to the challenges to educators was their limited knowledge and skills of ERTL (Naidoo et al., 2022). Naidoo et al. (2022) reported that ERTL was overwhelming and intimidating for educators, even with training, thus leading to uncertainty. These authors believed that although ERTL training was valuable, it was lacking in practical application and was more knowledge focused with poor theoretical coherence. These challenges with ERTL for educators had a substantial impact on their workload, which became exceptionally increased (Hlayisi, 2022; Khatib & Hlayisi, 2022; Naidoo et al., 2022). Naidoo et al. (2022) found that educators experienced substantial challenges in coping with the extra demands brought on by the ERTL student-centred approach that COVID-19 was demanding.

#### Practice

As far as challenges to practice were concerned, five themes were identified in the current review of papers in the SAJCD Special Issue (2022): (1) lack of sufficient knowledge and skills in telepractice and the use of advances in ICT for clinical practice by practising clinicians and students; (2) access and infrastructure challenges, for example, lack of appropriate equipment and limited access to data for connectivity; (3) high costs linked to telepractice; (4) increased or high workload; and (5) lack of and/or poor application of policies and regulations, for example, COVID-19 regulations that do not consider patients with special needs. Because of the COVID-19 regulations that were put in place to prevent the spread of the virus, as well as to manage those infected, important challenges were encountered with the traditional in-person models of service delivery. For clinical care to continue, adoption of innovative and mostly remote service delivery models became inevitable. Thus, telepractice became placed at the forefront of these models – therefore requiring knowledge and skills in this model. Khoza-Shangase (2022b) reported on the lack of sufficient knowledge and skills in telepractice by audiologists, while Madahana et al. (2022b) highlighted the lack of utilisation of AI for clinical application. This knowledge and application gap is a missed opportunity for clinical care in these fields during and beyond COVID-19. Additionally, Karrim et al. (2022) raised challenges with criteria, strategies and technical skills required for the utilisation of telerehabilitation by speech–language therapists and audiologists. For example, challenges were highlighted on telerehabilitation in children with autism spectrum disorder; middle ear assessments (Sebothoma & Khoza-Shangase, 2022); therapy for speech, language and swallowing following stroke (Tar-Mahomed & Kater, 2022); early communication intervention (Achmat & Gerber, 2022); neonatal feeding in neonatal intensive care units (Coutts et al., 2022); and geriatric audiology service provision in old age homes (De Andrade & Landman, 2022).

Challenges to practice created by COVID-19 were worsened by access and infrastructure challenges, as well as the related high infrastructural costs. Over and above infrastructure and system challenges directly related to curbing the spread of COVID-19 in the form of personal protective equipment (PPE) and infection prevention and control (IPC) measures (Achmat & Gerber, 2022), noteworthy other infrastructure and cost- related challenges were experienced by both professionals and patients (Khoza-Shangase, 2022b; McAllister et al., 2022; Tar-Mahomed & Kater, 2022). Access to the technology and its infrastructural support for innovative clinical service provision was raised as a serious barrier. This included (1) access to technological devices (e.g. computers, laptops, tablets and smartphones), with a lack of resources reported by Coutts et al. (2022); (2) connectivity in the form of access to Internet with good bandwidth; (3) stable power supply; and (4) technology-associated costs, including data costs (Khatib & Hlayisi, 2022; Khoza-Shangase, 2022a, 2022b; McAllister et al., 2022; Tar-Mahomed & Kater, 2022). In South Africa, a major challenge was maintaining a stable power supply with load-shedding, which is scheduled national electricity supply interruption. Khatib and Hlayisi (2022) raised limitations regarding available technologies such as technological compatibility of older hearing aids, while Khoza-Shangase (2022b) reported on challenges around technological abilities of patients, clinicians as well as patient-site facilitators (PSFs). Khatib and Hlayisi (2022) reported that patients were not ready for this model of service delivery, thus requiring deliberations around reliability of some online assessments. Tar-Mahomed and Kater (2022) highlighted that within the South African context, these challenges of technological access and supportive infrastructure are not the same across the health care sector. These authors reported that the private health care sector is better resourced than the public sector. However, regardless of the sector, in general, other health care services were prioritised over audiological services such as COVID-19, with financial constraints limiting the provision of audiological services during COVID-19 (De Andrade & Landman, 2022).

Furthermore, as far as challenges to practice are concerned, COVID-19 significantly increased the workload of clinicians (Balton et al., 2022; Hlayisi, 2022; Khatib & Hlayisi, 2022). Over and above the core function of providing clinical care, time devoted to establishing innovative modes of service provision, sourcing required resources that would allow for continuity of service provision during the pandemic, training and upskilling everyone on alternative service provision methods, as well as engagement in the national COVID-19 pandemic management were all added to clinicians’ workloads (Achmat & Gerber, 2022; Balton et al., 2022; Hlayisi, 2022; Karrim et al., 2022; Khatib & Hlayisi, 2022; Khoza-Shangase, 2022b; Nagdee et al., 2022). Balton et al. (2022) highlighted how such workload challenges were possibly influenced by a lack of or poor application of policies and regulations within the South African context. These authors argued that the response of the South African health care system to COVID-19 highlighted that the needs of vulnerable populations were not clearly taken into cognisance when regulations and policies to curb the pandemic were developed. Khoza-Shangase (2022b) underscored how regulations around reimbursement and registration to practice were barriers to the provision of clinical care during COVID-19 internationally and how these regulations require careful engagement. Karrim et al. (2022) raised restrictions around policies regarding the use of telepractice for assessment and treatment as another challenge. Khoza-Shangase (2022b) highlighted this challenge around policies and regulations as it relates to the use of PSFs, their training and their regulation as part of telepractice. This challenge stresses an urgent need for regularisation, standardisation and structurisation of PSFs in current training programmes as well as in the South African national health department’s human resource strategy.

### Evidence-based solutions for speech–language and hearing research, teaching and practice in the post-pandemic era

Valuable evidence-based solutions and future directions were identified from the papers included in the SAJCD Special Issue (2022).

#### Research

As far as research is concerned, the solutions emerged under four themes: (1) need for new and innovative approaches to research, (2) heightened focus on technological advances, (3) contextually relevant and responsive large-scale research in LMICs to address the key needs and (4) development and/or implementation of relevant policies. Khoza-Shangase et al. (2022) argued for new approaches to research that accommodate national public health emergencies. These authors also raised the importance of development of research infrastructure and workforce that will facilitate and sustain knowledge generation during and beyond pandemics. Furthermore, these authors highlighted maximising opportunities for remote working practices, which require improved use of ICT for research processes. Such approaches will enhance the quality of research designs (Khoza-Shangase, 2022a), including contextually relevant and responsive longitudinal as well as large-scale studies required in LMICs to address key needs for evidence-based practice (Coutts et al., 2022; Khan et al., 2022; Khatib & Hlayisi, 2022; Khoza-Shangase, 2022b; Masuku et al., 2022). Solutions must include considerations of, development and/or implementation of relevant policies so that researchers and participants are not harmed, research is supported and sustained, researchers are protected as employees and their mental health is looked after by research managers and research institutes. Details on the methods of support can be obtained in the individual articles of the Special Issue of SAJCD in 2022.

#### Teaching and learning

Solutions that were suggested to address teaching challenges fell under eight themes: (1) careful interrogation of core aspects of telepractice and inclusion of them in the undergraduate curriculum; (2) need for flexibility in terms of access to academic material; (3) need for robust technical infrastructure to support innovative teaching and learning; (4) comprehensive and enhanced student support across the years of study; (5) exploration of opportunities for international telesupervision; (6) need for advances in clinical training (ICT, fourth industrial revolution [4IR] and AI); (7) enhanced hybrid models for teaching and learning; and (8) deliberate reflection on personal challenges and contextual influences to build resilience and coping mechanisms (Khoza-Shangase, 2022b; Madahana et al., 2022a, 2022b; Naidoo et al., 2022).

With the overall recommendation of hybrid models of teaching and learning that include the use of ICT, simulations, teletraining and telesupervision (Barber & Sher, 2022; Masuku & Mupawose, 2022; McAllister et al., 2022; Nagdee et al., 2022), Naidoo et al. (2022) stressed that online platforms will facilitate closer and increased collaboration. This collaboration is between students and between students and educators, with increased opportunities for local and international collaborations. These authors further believed that online platforms for teaching and learning may raise opportunities to support students through social media platforms, thus increasing the amount of immediate and individual support offered to them. From their experience with telesupervision and online case-based learning for speech and language therapy students in Vietnam, McAllister et al. (2022) recommended telesupervision, where international therapists, working remotely and in partnership with local therapists, provide on-site supervision for students. This recommendation includes online case-based discussions as part of clinical training and online student group discussions, using case simulations with videos or avatars.

#### Practice

Clinical practice-related solution themes to COVID-19 that emerged from the articles in the Special Issue of SAJCD in 2022 were the following: (1) intensified research into contextually relevant and responsive solutions to guide best practice, (2) technologically intelligent clinical skills training, (3) the need to explore and/or formalise and regulate the use of task-shifting and PSFs during telepractice, (4) robust technical infrastructure to support telepractice, (5) use of hybrid rehabilitation models, (6) prioritisation of mental health services for both patients and their caregivers as well as health care workers and (7) the need for quality standards and best-practice models to be maintained (Coutts et al., 2022; De Andrade & Landman, 2022; Karrim et al., 2022; Khatib & Hlayisi, 2022; Khoza-Shangase, 2022a, 2022b; Madahana et al., 2022a, 2022b; Naidoo et al., 2022; Tar-Mahomed & Kater, 2022).

Some specific solutions emanating from the *foci* of the articles in the Special Issue of SAJCD in 2022 included (1) an increased need for careful audiological assessment as well as polymerase chain reaction testing for COVID-19 diagnosis in patients presenting with sudden unexplained cochleovestibular symptoms during COVID-19 (Khoza-Shangase, 2022a); (2) paradigm shift towards inclusion of AI and ML concepts in the current technologies being developed in South Africa as solutions for the SLH professions as the country transitions towards 4IR, such as the ML-linked solutions that are targeted towards bridging the communication gap between the normally hearing and people with hearing impairment (Madahana et al., 2022a, 2022b); (3) provision of and access to robust technical infrastructure to support all activities (Madahana et al., 2022a); and (4) the use of a hybrid approach to clinical service delivery (Khatib & Hlayisi, 2022). Khatib and Hlayisi (2022) highlighted that the utilisation of a hybrid approach including telepractice can lead to increased patient compliance, positive clinical benefits and positive participant experience, as well as cost-effective SLH services. Karrim et al. (2022) supported this approach and argued that despite the implementation difficulties associated with telepractice, the benefits far outweigh the challenges encountered.

Moreover, as far as practice solutions are concerned, Tar-Mahomed and Kater (2022) stressed the importance of considering factors such as linguistic and cultural diversity and socio-economic status. The socio-economic status issues include income, educational level and stable power supply, as well as access to ICT and its associated infrastructure as part of key solutions for practice challenges for both patients and service providers. Khoza-Shangase (2022b) added to these factors the crucial potential role of PSFs during telepractice within the South African context. This author argued that this solution carries the potential of enhancing contextual relevance in clinical service provision, if the PSFs are linguistically and culturally diverse and come from the communities where the patients reside. Furthermore, this solution, if properly planned and appropriately managed and regulated, can potentially create employment opportunities where paraprofessionals are employed in task-shifting roles as PSFs. However, this would require careful deliberations and planning around who can serve as PSFs, who will provide the training and the content and nature of the training they would require, as well as regulations around the quality standards and best-practice models that must be maintained. All the solutions presented thus far call for careful planning, research to establish evidence for best practice and proper and accurate record-keeping. Balton et al. (2022) raised the value of adaptability, becoming comfortable with uncertainty and maintaining open and transparent communication in clinical practice during and beyond COVID-19. Such qualities also require close collaboration within the various levels of the South African health care system to facilitate an appropriately response to the needs of patients during such a public health emergency. According to Balton et al. (2022), this hinges on commitment to compassionate leadership and staff well-being.

### Issues that require further investigation for improved readiness for future pandemics

Current evidence emerging from the Special Issue of SAJCD in 2022 reviewed articles suggests a clear need for research in the following areas:

1. Research is needed on the potential impact of remote (online) teaching and learning, as well as telepractice.
2. Studies should be conducted on the use of simulations as well as writing-intensive approaches to teaching for clinical training.
3. Rigorous scientific investigation of the effects of COVID-19 on SLH services that includes specific evaluation of the many different areas of communication disorders that are clinically diagnosed and treated by SLH professionals.
4. Research into the use of and development of AI and ML technologies in SLH research, teaching and clinical practice.
5. Research into technologies for speech-to-text conversion in South African languages other than English.
6. Exploration of remote international clinical supervision.
7. Research into hearing health care human resource policies and planning, hearing health care labour market needs and capacity, as well as hearing health care service delivery and potential for growth in the South African context after COVID-19.
8. Reviews of policies and regulations around COVID-19 and similar public health emergencies and how these affect SLH professions and the vulnerable populations they serve.
9. Efficacy studies on the use of telepractice in SLH service provision.The effect of PPE and IPC on SLH services.

The above-listed areas of future research direction and recommendations in this COVID-19-themed Special Issue of SAJCD in 2022 raise important concerns for future enquiry and developments for the SLH professions from LMICs, which may also have global relevance. Each manuscript provides specific, more detailed future research implications, contextualised within each paper.

## Limitations

Although the qualitative scoping review approach adopted in the current review was appropriate for the purpose and questions of the current study, findings are limited to data presented in the manuscripts included in the Special Issue of SAJCD in 2022 on COVID-19 research. This limitation must therefore be considered when interpreting the findings presented, as findings cannot be generalised and cannot be assumed to represent the comprehensive body of knowledge in the area that the Special Issue of SAJCD in 2022 specifically focused on, and challenges, solutions and areas for further investigation presented in this article are only reflective of those covered in the 21 manuscripts included in this Special Issue of SAJCD in 2022.

## Conclusion

This special issue entitled ‘The impact of COVID-19 in SLH professions in LMICs: Challenges and opportunities explored’ revealed important challenges encountered by LMICs in terms of research, teaching and practice. Although specific to the SLH professions, findings presented in this article may have global applicability within broader health care research, teaching and training. The challenges identified, as presented under themes, call for strategic planning, innovative and flexible solutions and sensitivity to contextual realities in SLH research, teaching and practice. These solutions must be driven by evidence developed from research that adheres to rigorous scientific standards, with enhanced appreciation of what ICT, AI and 4IR have to offer to these professions. Some of the issues highlighted by the Special Issue of SAJCD in 2022 that require further investigation for improved readiness for future pandemics, which have been detailed in this article, require engagement and deliberate planning for SLH professions to be better prepared not only for public health emergencies but also for re-imagining SLH practice within LMICs.
